# A case of resected hepatocellular carcinoma with gallbladder metastasis

**DOI:** 10.1186/s40792-021-01222-7

**Published:** 2021-06-17

**Authors:** Takaaki Hanazawa, Yasuyuki Fukami, Takaaki Osawa, Shintaro Kurahashi, Tatsuki Matsumura, Takuya Saito, Shunichiro Komatsu, Kenitiro Kaneko, Toyonori Tsuzuki, Tsuyoshi Sano

**Affiliations:** 1 Division of Gastroenterological Surgery, Department of Surgery, Aichi Medical University, 1-1 Yazakokarimata, Nagakute, Aichi 480-1195 Japan; 2 Department of Surgical Pathology, Aichi Medical University, Nagakute, Aichi Japan

**Keywords:** Hepatocellular carcinoma, Gallbladder, Metastasis, Liver resection

## Abstract

**Background:**

Advanced hepatocellular carcinoma (HCC) can often spread as intrahepatic metastases. Extrahepatic metastasis (e.g., lung, lymph nodes, and bones) is rare, and gallbladder metastasis from HCC is extremely rare.

**Case presentation:**

A 66-year-old woman who presented with right hypochondrial pain was referred to our hospital for further examination of a liver tumor. The blood chemistry data showed elevated levels of serum α-fetoprotein (AFP) (3730 ng/mL), protein induced by vitamin K absence or antagonist II (PIVKA-II) (130 mAU/mL), and carcinoembryonic antigen (CEA) (358.6 ng/mL). Hepatitis B surface antigen and hepatitis C virus antibody were negative. Dynamic computed tomography (CT) showed a tumor measuring 12 × 7 cm in the right lobe of the liver. This tumor was contrast-enhanced in the hepatic arterial phase and then became less dense than the liver parenchyma in the portal phase. A well-enhanced tumor was found in the gallbladder. No regional lymph nodes were enlarged. Contrast-enhanced magnetic resonance imaging (MRI) demonstrated that the liver tumor showed a pattern of early enhancement and washout. The gallbladder tumor was also detected as an enhanced mass. Endoscopic retrograde cholangiography (ERC) showed compression of the left hepatic duct due to the liver tumor. The patient was diagnosed with simultaneous HCC and gallbladder cancer. Right hepatic trisectionectomy and caudate lobectomy with extrahepatic bile duct resection were performed. Histopathological examination of the resected liver specimen showed a poorly differentiated HCC cell component with a trabecular and solid growth, and diffuse invasion of the portal vein. The same tumor cells were found in the gallbladder, but no continuity with the liver tumor was identified. Immunohistochemistry of the liver tumor and gallbladder was positive for AFP, Glypican 3, and CK7, and negative for CK19. The final pathological diagnosis was the gallbladder metastasis from HCC. A follow-up diagnostic image 33 months after surgery showed a mass in the upper lobe of the left lung. The patient underwent left upper lobectomy. Postoperative pathology revealed that the lung lesion was a metastasis of HCC. The patient was still alive with lung metastasis and was being treated with a molecular-targeting drug in good health 42 months after the initial surgery.

**Conclusions:**

The standard treatment for advanced HCC with extrahepatic metastases is molecularly targeted drugs, but surgery is also an option if the lesion can be resected en bloc without remnants.

## Background

Advanced hepatocellular carcinoma (HCC) can spread as intrahepatic metastases more easily than to extrahepatic metastases (e.g., lung, lymph nodes, and bones). Gallbladder metastasis from HCC is extremely rare. Here, we report the long-term palliation of HCC with gallbladder metastasis, treated with aggressive surgery.

## Case presentation

**Fig. 1:**
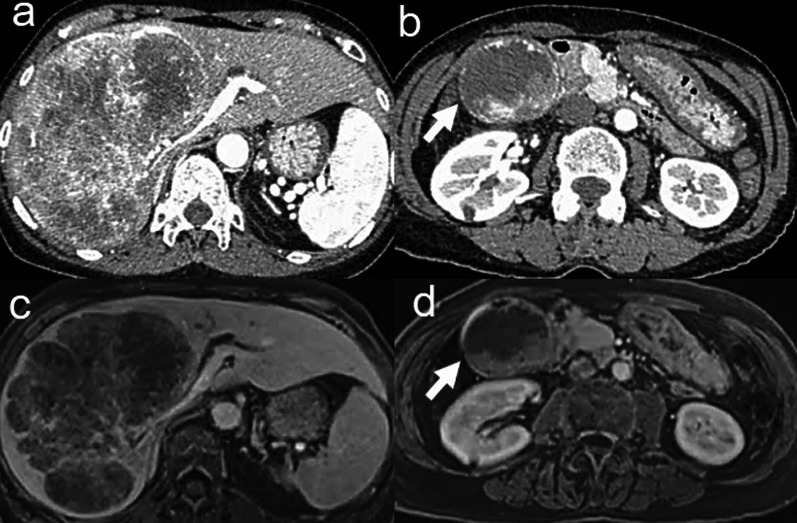
**a** Dynamic computed tomography (CT) shows a tumor measuring 12 × 7 cm in diameter in the right lobe of the liver. **b** A well-enhanced tumor is found in the gallbladder. **c** Contrast-enhanced magnetic resonance imaging (MRI) demonstrated the liver tumor. **d** The gallbladder tumor is also detected as an enhanced mass arrow

**Fig. 2:**
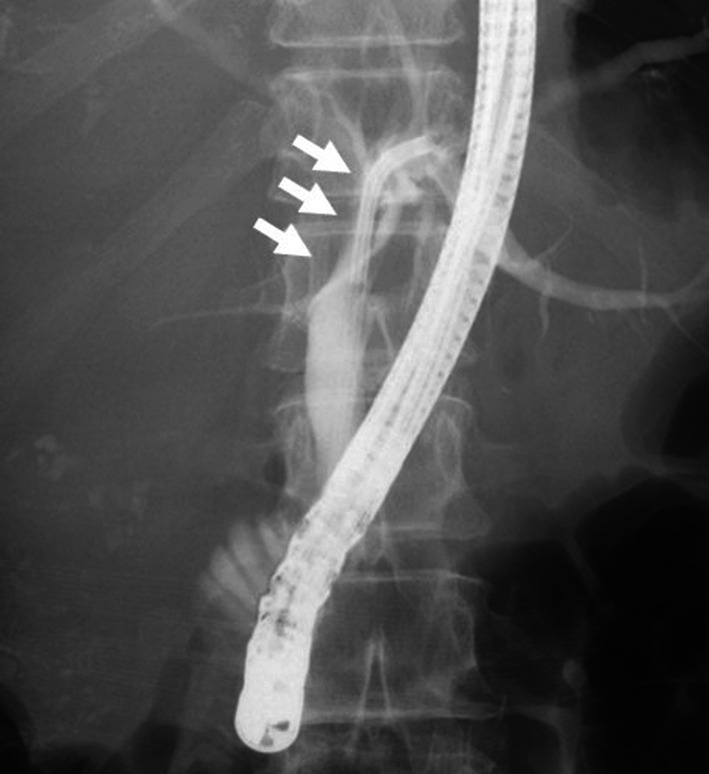
Endoscopic retrograde cholangiography (ERC) shows compression of the left hepatic duct with arrows

A 66-year-old woman with right hypochondrial pain was referred to our hospital for further examination of a liver tumor. The blood chemistry data showed elevated white blood cells (11,700/mm^3^), C-reactive protein (1.36 mg/dL), aspartate aminotransferase (61 U/L), alanine transferase (50 U/L), serum α-fetoprotein (AFP) (3730 ng/mL), protein induced by vitamin K absence or antagonist II (PIVKA-II) (130 mAU/mL), and carcinoembryonic antigen (CEA) (358.6 ng/mL). Hepatitis B surface antigen and hepatitis C virus antibody were negative. Dynamic computed tomography (CT) showed a tumor measuring 12 × 7 cm in diameter in the right lobe of the liver (Fig. 1a). This tumor showed contrast enhancement in the hepatic arterial phase and then became less dense than the liver parenchyma in the portal phase. In addition, a well-enhanced tumor was found in the gallbladder (Fig. 1b). No regional lymph nodes were enlarged. Contrast-enhanced magnetic resonance imaging (MRI) demonstrated that the liver tumor showed a pattern of early enhancement and late washout (Fig. 1c). The gallbladder tumor was also detected as an enhanced mass (Fig. 1d). Endoscopic retrograde cholangiography (ERC) showed that compression of the left hepatic duct due to the liver tumor (Fig. 2). In addition, preoperative CT revealed that the gallbladder tumor was suspected to have invaded the common bile duct. If the gallbladder tumor also invades the common bile duct, extrahepatic bile duct resection is necessary. Therefore, a right hepatic trisectionectomy and caudate lobectomy with extrahepatic bile duct resection including regional lymph adenectomy were planned in this case. The patient was diagnosed with HCC (T3N0M0 stage III according to the International Union against Cancer (UICC) classification) and gallbladder cancer (T2N0M0 stage II according to the UICC classification).

**Fig. 3:**
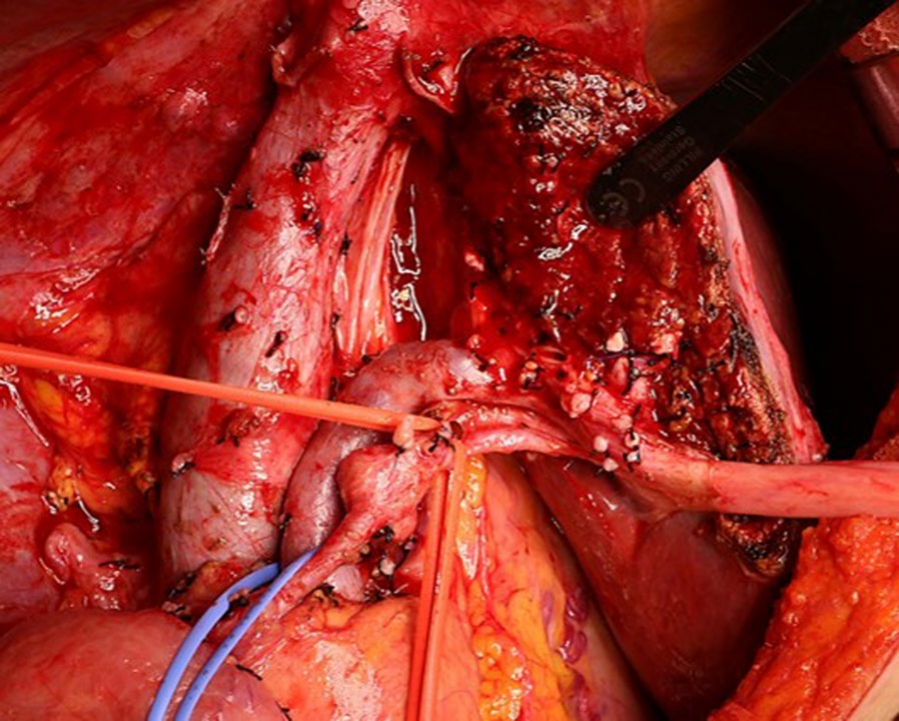
A right hepatic trisectionectomy and caudate lobectomy with extrahepatic bile duct resection including regional lymph adenectomy for gallbladder cancer are completed

**Fig. 4:**
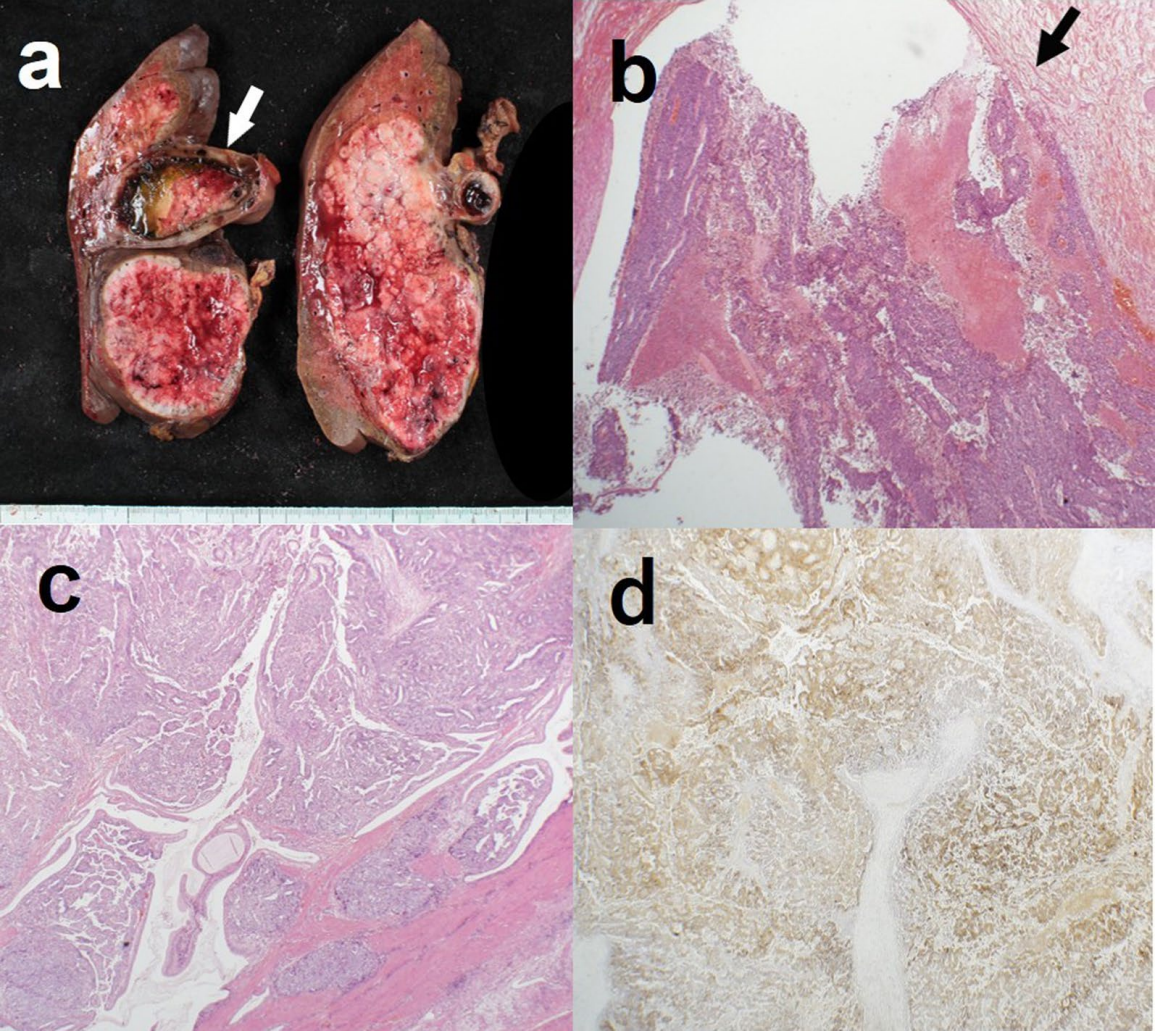
**a** Cut surface of the resected specimens shows multinodular fused lesions in the liver and a papillary tumor in the gallbladder with arrow. **b** Liver specimen shows a poorly differentiated HCC cells component with a trabecular and solid growth, and diffuse invasion of the portal vein including with arrow (× 20, H&E stain). **c** The tumor cells in the gallbladder show trabecular pattern indicating HCC (× 40, H&E stain). **d** Immunostaining of the gallbladder tumor is positive for AFP (× 20, H&E stain)

The preoperative liver function in this patient was well maintained at Child–Pugh A, and the indocyanine green retention rate at 15 min was 7.3%. The remnant liver volume with a right hepatic trisectionectomy was 381 mL (42.9%). Therefore, a right hepatic trisectionectomy and caudate lobectomy with extrahepatic bile duct resection including regional lymph adenectomy were performed (Fig. 3). Intraoperative findings also could not deny direct invasion to the common bile duct. The operative time was 486 min, and blood loss was 400 mL. The postoperative course was uneventful and the patient was discharged on postoperative day 25. Histopathological examination of the resected liver specimen (Fig. 4a) showed a poorly differentiated HCC cells component with a trabecular and solid growth, and diffuse invasion of the portal vein (Fig. 4b). As for the extrahepatic bile ducts, lymphatic invasion was observed around the bile ducts. The same tumor cells were found in the gallbladder, but no continuity with the liver was identified (Fig. 4c). The depth of the gallbladder tumor in this case was invasion to the subserosa layer on the peritoneal side. It corresponded to T2b in the UICC classification of gallbladder cancer. Immunohistochemical examination of the liver tumor and gallbladder tumor detected AFP (Fig. 4d), Glypican 3, and CK7, but not CK19. The final pathological diagnosis was the gallbladder metastasis from HCC (T4N0M1 stage IV according to the UICC classification). A follow-up diagnostic image 33 months after surgery showed a mass in the upper lobe of the left lung. Positron emission tomography (PET)/CT showed accumulation, so the patient underwent left upper lobectomy. Postoperative pathology revealed that the lung lesion was a metastasis of HCC. The patient was still alive with lung metastasis and was being treated with a molecular-targeting drug in good health 42 months after the initial surgery.

## Discussion

HCC is one of the most common malignancies, responsible for an estimated 745,000 deaths per year [1, 2]. The Barcelona Clinic Liver Cancer (BCLC) staging system is widely used as one of the treatment guidelines for HCC in Western countries [3, 4]. According to the BCLC classification system, surgical resection is indicated only in some patients with early-stage HCC and satisfactory liver function. HCC with extrahepatic metastasis are considered intermediate-stage HCC, for which systemic therapy (e.g., sorafenib) is recommended. However, in the setting of clinical practice, patients with limited extrahepatic metastasis from HCC are often referred to surgical teams to be evaluated for curative resection. A major limitation of the BCLC staging system is the lack of external validation. Given these conflicting recommendations, the efficacy of surgical resection for treating HCC patients needs to be clarified.

The most frequent site of extrahepatic metastasis is the lung followed by the lymph nodes, bone, and adrenal glands. Uchino et al. demonstrated that the major cause of death in patients with HCC who have extrahepatic metastasis is the progression of intrahepatic HCC lesions and that extrahepatic metastasis is not often the direct cause of death. They reported that the median survival after diagnosis of extrahepatic metastasis was 8.1 months [5]. Gallbladder metastasis from HCC is extremely rare, so it is difficult to determine the indications for surgical resection of it. In addition, as in this particular case, the differential diagnosis of double primary cancer (i.e., HCC and gallbladder cancer) or gallbladder metastasis from HCC is difficult even with recent advances in preoperative diagnostic images. Although tumor was detected as an enhanced mass in the gallbladder in the present case, there are no specific findings in the imaging modality of gallbladder metastasis from HCC [6, 7].

Murakami et al. reported that among 393 HCC patients who underwent hepatectomy with cholecystectomy, eight (1.8%) had gallbladder metastasis without other distant metastases. These patients had closely related portal vein tumor thrombosis (PVTT), so they recommended surgical resection aiming at long-term survival [8]. Wakasugi et al. reviewed 16 cases of resected gallbladder metastasis from HCC. Thirteen of the patients had a primary tumor in Couinaud’s segment 4 or 5, and 11 patients had PVTT [9]. Nakashima et al. suggested four possible routes to the gallbladder from HCC: the most likely is the hematogenous route via the portal venous system, the second is the lymphogenous route, the third is direct invasion of HCC from the liver, and the fourth is gallbladder metastasis with peritoneal dissemination of HCC [10]. In our case, we think that the hematogenous metastatic route was most likely given the pathological observations: HCC with massive portal vein invasion and no continuity between the liver and gallbladder tumors.

There are only five reported patients, including our patient, who survived over 2 years after surgical resection for gallbladder metastasis from HCC (Table 1) [8, 11, 12]. No characteristic profiles other than curative resection were found to achieve long-term survival. There is no consensus on the treatment of extrahepatic metastasis of HCC. In general, the indications for resection of the extrahepatic metastasis of HCC are limited because of the poor postresectional survival rate [13]. There are few reports of the resection of metastatic HCC in sites other than the lung, but metastasectomy appears to prolong survival [14]. Surgical resection could contribute to long‐term survival in cases of isolated gallbladder metastasis. Considering the current lack of effective medical treatment for extrahepatic metastases, resection of HCC with gallbladder metastases, prior to introduction of molecular-targeting agents is still a potent treatment of choice for long-term palliation.

**Table 1:** Surgical resection for gallbladder metastasis from HCC

Case	Author	Year	Age	Gender	HCC location^a^	HCC size (cm)	Operation	Postoperative therapy	Prognosis
1	Maruno	1994	73	Male	4	4.8	Lt. hepatectomy	Omentectomy, TAE	32 months alive
2	Nishida	1997	48	Male	4/5	NA	Wedge resection of the gallbladder bed	NA	NA
3	Lane	2002	78	Male	Right lobe	NA	Wedge resection of the gallbladder bed	NA	NA
4	Terashima	2007	49	Male	5/6/7/8	10.7	Wedge resection of the gallbladder bed	Hepatectomy, TAI	13 months alive
5	Ando	2009	75	Male	NA	NA	Wedge resection of the gallbladder bed	NA	NA
6	Murakami	2010	53	Male	7/8	14	Rt. hepatectomy	FAIT	63 months alive
7	Murakami		61	Male	5/8	9.5	Rt. hepatectomy	FAIT	4 months alive
8	Murakami		79	Male	2/3/4	13	Lt. hepatectomy	FAIT	6 months dead
9	Murakami		47	Male	4	6.5	Lt. hepatectomy	FAIT	54 months dead
10	Murakami		47	Male	2/3/4	13	Lt. hepatectomy	FAIT	9 months dead
11	Murakami		32	Male	5/6/7/8	15	Rt. hepatectomy	FAIT	3 months dead
12	Murakami		74	Male	5/6	5	Rt. hepatectomy	None	5 months dead
13	Murakami		66	Male	5/8	3.5	Rt. anterior sectionectomy	None	6 months dead
14	Monden	2011	66	Male	5/8	NA	Wedge resection of the gallbladder bed	None	10 months alive
15	Kanzaki	2011	48	Male	5	1.3	Wedge resection of the gallbladder bed	None	24 months alive
16	Wakasugi	2012	74	Male	1/5/6/7/8	8.8	Wedge resection of the gallbladder bed	Sorafenib	2 months dead
17	Our case	2020	66	Female	5/6/7/8	16	Rt. trisectionectomy	Left upper lobectomy, renvatinib	42 months alive

*HCC* hepatocellular carcinoma, *GB* gallbladder, *FAIT* intra-arterial infusion of 5-FU and subcutaneous interferon-alpha injection therapy

^a^Numerals indicating Couinaud’s segment of the liver

## Conclusions

We report a case of radical resection of HCC with gallbladder metastases. The standard treatment for advanced HCC with extrahepatic metastases is molecularly targeted drugs, but surgery is also considered as an option if the lesion can be resected en bloc without remnants.

## Data Availability

The datasets supporting the conclusions of this article are included within the article and its additional files.
